# Development of an SSR-based genetic map in sesame and identification of quantitative trait loci associated with charcoal rot resistance

**DOI:** 10.1038/s41598-017-08858-2

**Published:** 2017-08-21

**Authors:** Linhai Wang, Yanxin Zhang, Xiaodong Zhu, Xiaofeng Zhu, Donghua Li, Xianmei Zhang, Yuan Gao, Guobin Xiao, Xin Wei, Xiurong zhang

**Affiliations:** 1 Oil Crops Research Institute of the Chinese Academy of Agricultural Sciences, Key Laboratory of Biology and Genetic Improvement of Oil Crops of the Ministry of Agriculture, Wuhan, 430062 China; 2 Luohe Academy of Agricultural Sciences, Luohe, 462300 China; 3 Jiangxi Institute of Red Soil, Jinxian, 331717 China

## Abstract

Sesame is prized for its oil. Genetic improvement of sesame can be enhanced through marker-assisted breeding. However, few simple sequence repeat (SSR) markers and SSR-based genetic maps were available in sesame. In this study, 7,357 SSR markers were developed from the sesame genome and transcriptomes, and a genetic map was constructed by generating 424 novel polymorphic markers and using a cross population with 548 recombinant inbred lines (RIL). The genetic map had 13 linkage groups, equalling the number of sesame chromosomes. The linkage groups ranged in size from 113.6 to 179.9 centimorgans (cM), with a mean value of 143.8 cM over a total length of 1869.8 cM. Fourteen quantitative trait loci (QTL) for sesame charcoal rot disease resistance were detected, with contribution rates of 3–14.16% in four field environments; ~60% of the QTL were located within 5 cM at 95% confidence interval. The QTL with the highest phenotype contribution rate (*qCRR12*.*2*) and those detected in different environments (*qCRR8*.*2* and *qCRR8*.*3*) were used to predict candidate disease response genes. The new SSR-based genetic map and 14 novel QTLs for charcoal rot disease resistance will facilitate the mapping of agronomic traits and marker-assisted selection breeding in sesame.

## Introduction

Sesame (*Sesamum indicum L*.), a member of the family *Pedaliaceae* ^1^, is grown widely in tropical and subtropical areas^2^. It is considered one of the first oil seed crops known to humanity, and its domestication has been dated to 3,050−3,500 BC based on charred sesame remains recovered from archaeological excavations^3, 4^. Sesame is prized for its high oil content (~55% in the seeds), which exceeds that from other oilseed crops such as rapeseed, peanut, soybean, and sunflower. It is popular among consumers of east and south Asian countries for its high-quality oil and the broad utility of its seeds. Sesame seeds contain special anti-oxidative furofuran lignans such as sesamin and sesamolin^5, 6^, which demonstrate pharmacological properties thought to decrease blood lipids^7^ and lower cholesterol levels^8^. Despite these elite characteristics, studies on sesame remain insufficient compared with other crops, contributing to its low and unstable yield capacity^9^. According to the Food and Agriculture Organisation (FAO), the average yield of sesame was only 576 kg/ha in 2014 (http://www.fao.org), ranking second from lowest among 22 oil crops worldwide. Solid genetic studies are necessary to improve its resistance to biotic stress from fungal and bacterial diseases such as stem charcoal rot, *Fusarium* wilt, and powdery mildew.

Charcoal rot is one of the most damaging diseases, typically inducing 30% decrease or even complete loss of sesame crops. The disease is caused by *Macrophomina phaseolina* (Tassi) Goid. *M*., a seed- and soil-borne fungal pathogen that can infect nearly 500 plant species in more than 100 families^10–13^, including important crops such as peanut, cabbage, pepper, chickpea, soybean, sunflower, sweet potato, alfalfa, sesame, potato, sorghum, wheat, and corn^11, 14–16^. In sesame, charcoal rot occurs mainly at the end stage of flowering to maturity, with black spots initiating from the root or stem during hot and dry weather or under unfavourable environmental stresses^17–19^. Controlling the disease in a safe and efficient way is a current pivotal problem facing plant pathologist, geneticist, and breeders. Although breeding cultivars with integrated resistant genes is expected to be fundamentally the best choice, progress in genetic improvement efforts has been slow due to the lack of information regarding the gene–for–gene relationship between sesame and *M*. *phaseolina* fungus^20^.

Genetic mapping provides the foundation for genetic study, especially for discovering and manipulating the loci or genes underlying simple and complex traits in crop plants^21, 22^. The construction of genetic linkage maps, QTL mapping, and evolutionary analyses performed in standard molecular biology laboratories have provided SSR markers the primary choice for marker-assisted selection (MAS), based largely on the properties of co-dominance, reproducibility, and relative abundance in complete genomes^23–25^. However, molecular genetic research in sesame had lagged for decades, and some SSR markers were developed only recently^26–29^. The first map for sesame was constructed by Wei *et al*. based on amplified fragment length polymorphisms^30^ and was last updated in 2013.

Since few SSRs have been validated, genetic maps and gene mapping have been hampered in sesame. Several SNP-based maps were constructed by restriction-site-associated DNA sequencing (RAD-seq) and specific length amplified fragment sequencing (SLAF-seq) technologies using next generation sequencing platforms in recent years^31–33^. However, such genetic maps and SNP tags are not easy to be used by most sesame researchers in different molecular laboratories, because of the lack of available sequence information and special instruments.

The present study was designed to develop a greater number of SSR markers based on sesame genome and transcriptome sequences^34, 35^, and construct a genetic map with these co-dominant markers. Furthermore, the loci associated with sesame charcoal rot resistance were screened. The SSR-based genetic map will provide an essential and effective tool for QTL mapping of genes to identify various traits in sesame, facilitating future gene exploration and discovery of elite sesame cultivars for more productive breeding.

## Results and Discussion

### Polymorphic genomic-SSR development

A total of 110,495 genomic-SSR loci were detected in the sesame genome using the microsatellite identification tool (MISA) software. Of these, 39.1% were mono-nucleotides, 34.3% were di-nucleotides, 17.7% were tri-nucleotides, and 27.2% were compound SSRs. Relying on a previously published sesame transcriptome, 7,702 cDNA-SSR loci were investigated^28^. Here, 5,587 genomic-SSRs and 1,770 cDNA-SSRs were selected for designing primer pairs to synthesise markers. PCR analysis showed that 498 of the markers were polymorphic between the parents, ZZM2748 and Zhongzhi No. 13, accounting for 6.8% of the total synthesised markers. As these markers were developed based on the sesame genome, such differences might represent general polymorphisms of sesame SSR loci. However, SSRs with varying repeat units differed in their polymorphisms. The highest levels of polymorphisms were found with dinucleotide repeat units (11.9%), followed by compound (7.8%) and mononucleotide (7.4%) repeat units. Tetra- and pentanucleotide repeat unit SSRs showed lower polymorphic rates, <2%.

### SSR-based genetic map construction

**Figure 1:**
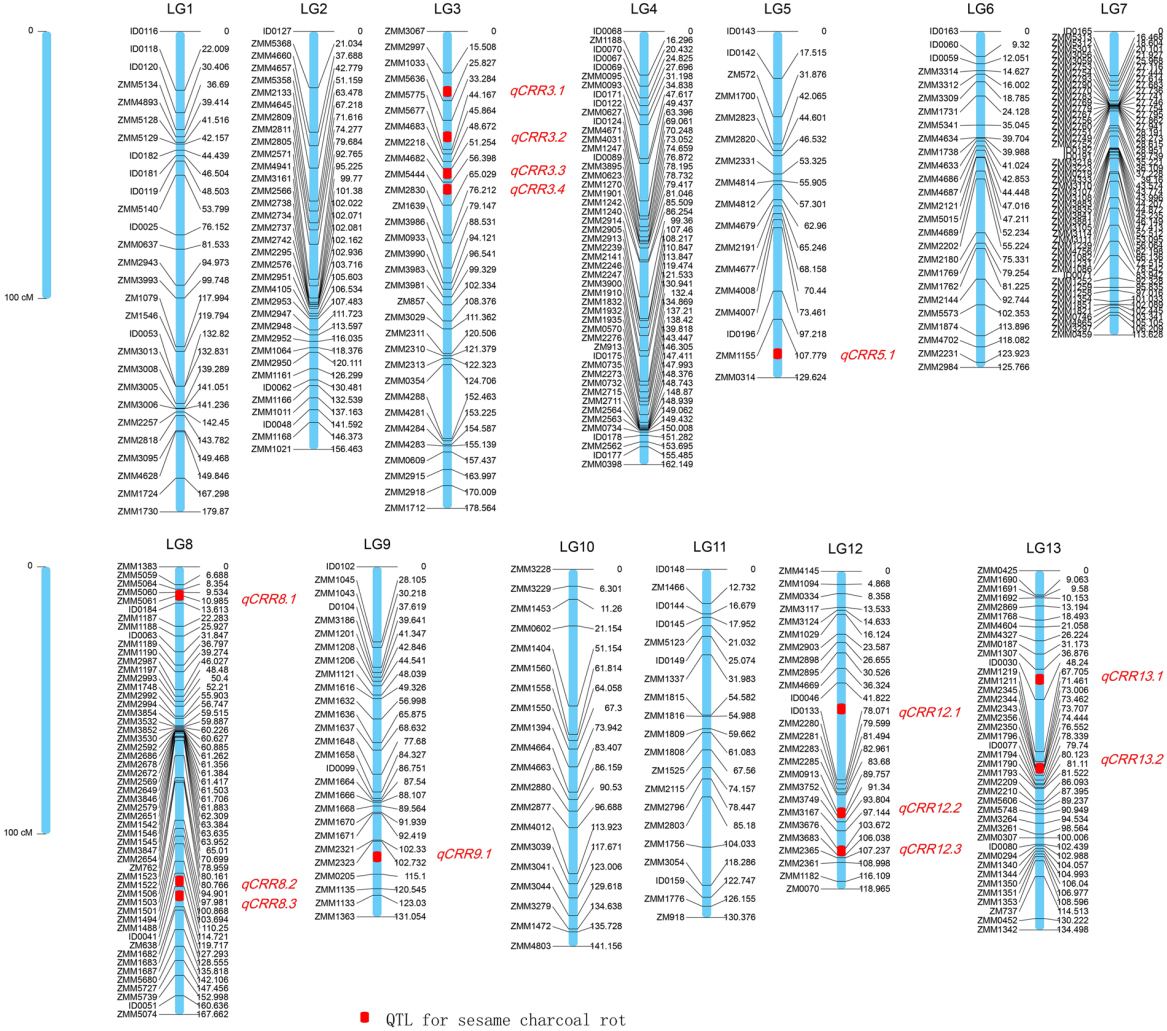
The simple sequence repeat (SSR)-based genetic map of the sesame genome and the mapped quantitative trait loci (QTL). Positions of the QTL for sesame charcoal rot response are indicated with red rectangles centred at the peak of each location.

All 498 polymorphic SSR markers were used to genotype the 548 RILs generated from the cross between ZZM2748 and Zhongzhi No. 13. After filtering out markers that lacked polymorphic alleles and those with significantly distorted segregation ratios (P < 0.01)^36, 37^, the remaining 462 markers were used to construct a genetic map using Joinmap 4.0. Finally, 424 SSR markers were mapped to the genetic map and distributed into 13 linkage groups (LG). All the 424 mapped markers were newly developed and published (Table S1). The LGs were numbered from LG1 to LG13 (Table 1, Fig. 1) and corresponded to the 13 assembled pseudomolecule chromosomes of sesame^38^.

**Table 1:** Summary of the sesame genetic map constructed with 424 SSR markers.

Linkage group	No. of markers	Map length (cM)	Maximum interval (cM)	Average interval (cM)	Physical length (Mb)	Recombination rates (cM/Mb)
LG1	28	179.9	22.4	6.7	20.3	8.9
LG2	35	156.5	21	4.6	18.4	8.5
LG3	31	178.6	27.8	6.0	25.9	6.9
LG4	49	162.1	16.3	3.4	20.6	7.9
LG5	17	129.6	23.8	8.1	16.6	7.8
LG6	26	125.8	20.1	5.0	26.0	4.8
LG7	52	113.6	16.5	2.2	16.8	6.8
LG8	53	167.7	14.1	3.2	26.2	6.4
LG9	27	131.1	28.1	5.0	22.9	5.7
LG10	20	141.2	30	7.4	19.5	7.2
LG11	20	130.4	22.6	6.9	14.1	9.3
LG12	26	119.0	36.2	4.8	16.3	7.3
LG13	40	134.5	19.5	3.4	16.5	8.2
Total	424	1869.8			259.7

The lengths of the 13 LGs ranged from 113.6 to 179.9 cM, with a mean value of 143.8 cM, and the resulting map length was 1869.8 cM in total. The number of markers in each LG ranged from 17 (LG5) to 53 (LG8). The interval distances between adjacent markers varied from 0.1 to 36.2 cM, with a mean interval distance of 5.1 cM across different LGs; 81.4% of markers showed interval distances less than 10 cM relative to adjacent markers. The highest marker density was observed in LG7 (an average of 2.2 cM between adjacent markers), followed by LG8 (3.2 cM) (Fig. 1); LG5 showed the lowest density average (8.1 cM). Obviously, the total marker number in the map was less than those presented in previously published genetic maps that were constructed using next-generation sequencing technology, such as RAD-seq (1,230 and 1,522)^31, 38^ and SLAF-seq (1,233)^32^. As few SSR-based genetic maps were available previously and a large number of markers need to be designed and screened for polymorphisms with proper segregation of the population for genotyping, the construction of such a SSR-based genetic map was very time consuming, which lasted for 5 years. However, considering the small and diploid genome of sesame (357 Mb), the current map is competent for genetic analysis including genetic or QTL mapping.

### Mapping the QTL associated with charcoal rot resistance

**Figure 2:**
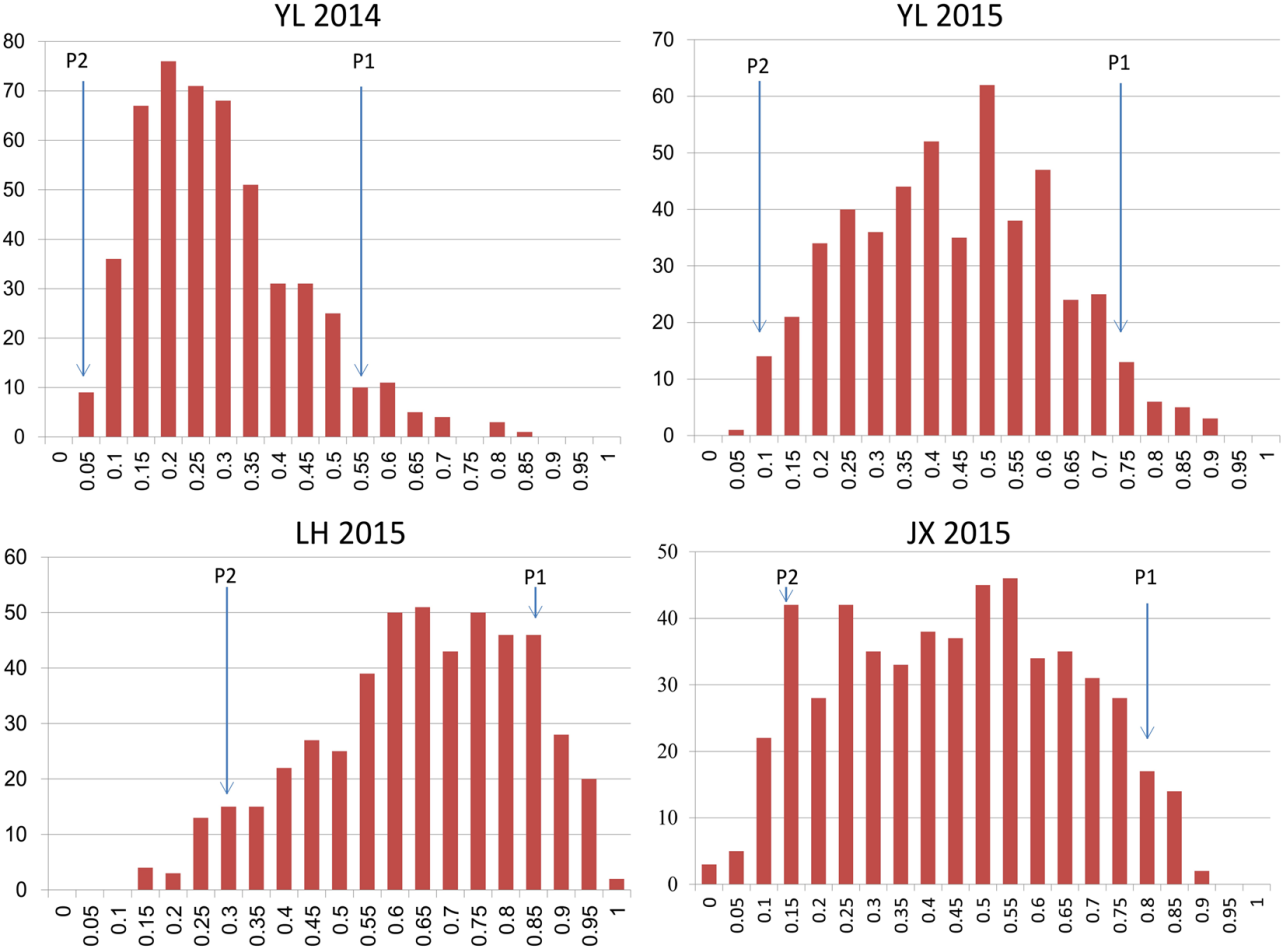
Disease index (DI) variations across different environments in the RIL population. DI, the charcoal rot disease index of the recombinant inbred lines (RIL). YL 2014, Yangluo environment in 2014; YL 2015, Yangluo environment in 2015; JX 2015, Jinxian environment in 2015; LH 2015, Luohe environment in 2015.

**Figure 3:**
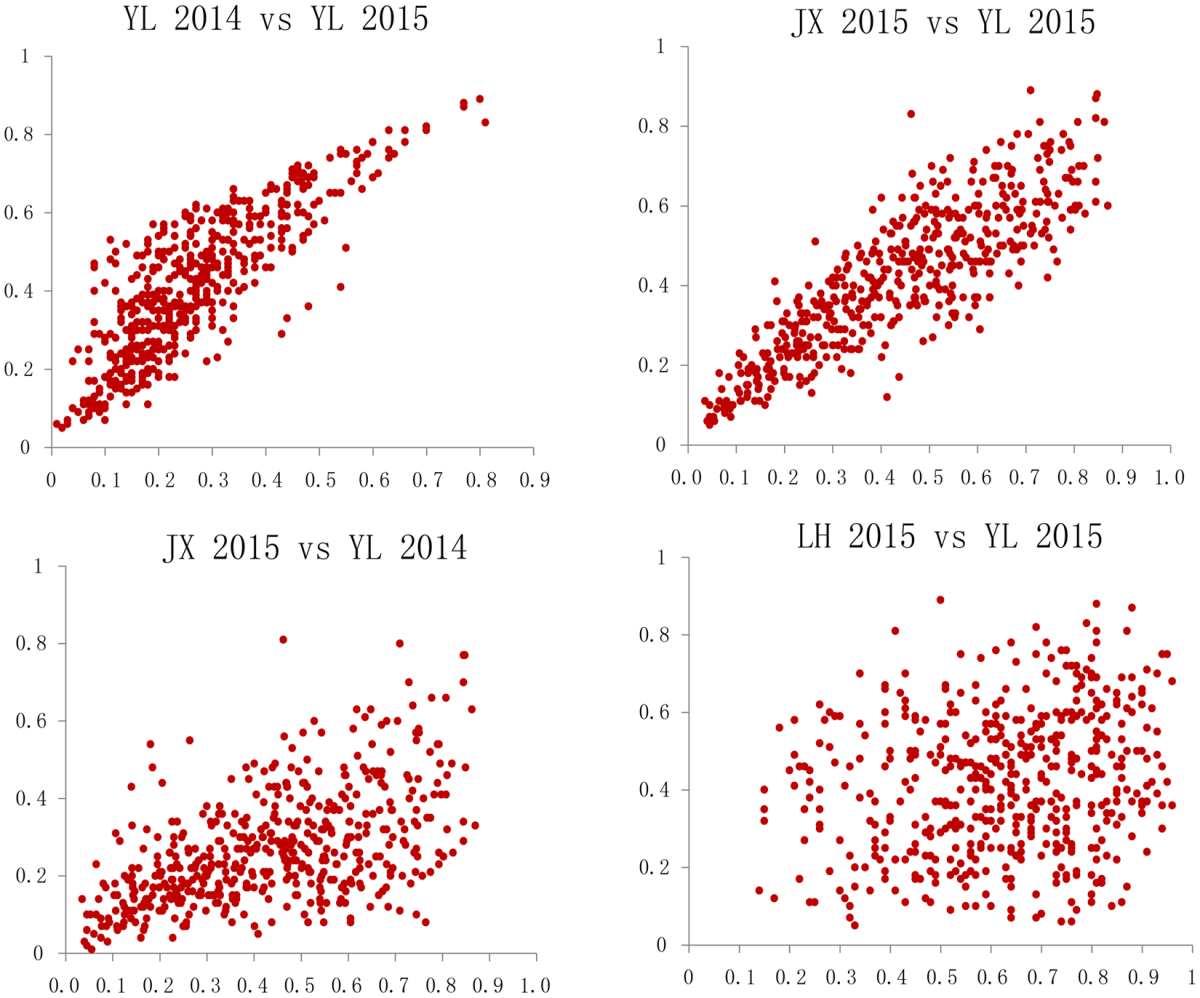
Scatter plots of the co-relationships between the DIs from the four locations. YL 2014, Yangluo environment in 2014; YL 2015, Yangluo environment in 2015; JX 2015, Jinxian environment in 2015; LH 2015, Luohe environment in 2015.

The charcoal rot response of the 548 RILs and the two parents was evaluated in four growing environments including Luohe (2015), Jinxian (2015), and Yangluo (2014 and 2015). Jinxian and Yangluo are in southern China along the Yangtze River, and they typically suffered from serious charcoal rot disease during the sesame planting seasons. Luohe, in northern China, also experienced serious charcoal rot disease during the sesame planting season. In 2014, the disease index (DI) at the Yangluo site ranged from 0.01 to 0.81 and averaged 0.31. In 2015, the ranges of DI in the three sites were 0.14–0.96 (Luohe, average 0.60), 0.0–0.87 (Jinxian, average 0.42), and 0.05–0.89 (Yangluo, average 0.42) respectively (Fig. 2). The DIs of Zhongzhi No. 13 were generally <0.15, whereas ZZM2748 displayed DIs >0.50 in different environments. ANOVA revealed significant differences (*P* = 0.01) in DIs among RILs, environments, and line × environment interactions, but not among environmental replicates (Table S2). The DIs from the four environments showed that Yangluo and Jinxian were highly correlated, but they all showed low correlations with Luohe. Those findings reveal the diversities among *M*. *phaseolinas* pathogenic races in southern and northern China, as well as among environmental conditions (Fig. 3).

Using the composite interval mapping method implemented in Windows QTL Cartographer 2.5 software (Microsoft, Inc., Redmond, WA, USA)^39^, 14 QTLs were found to be significantly associated with sesame charcoal rot disease resistance, with contribution rates of 3–14.16% (mean, 6.95%; Table 2, Fig. 1). A previous study showed enhanced infection of *M*. *phaseolina* when soil water was deficient, and several overlapped or pleiotropic QTLs for drought tolerance and charcoal rot resistance were reported in other crops^40, 41^. Sesame is generally drought tolerant, as it originated from the tropical regions of Africa or India^42^, and several of these loci may function in drought tolerance. However, such cases require further investigation, as no genes or QTLs for sesame drought-tolerance have yet been identified.

**Table 2:** Mapped QTLs* associated with sesame charcoal rot resistance.

Trait	Chrom/LG	Position (cM)	Flanking markers	Additive effect	R2	YL2014	YL2015	JX2015	LH2015
*qCRR3*.*1*	3	24.50	ZMM2997~ZMM1033	0.03	0.03	Y	Y	Y	
*qCRR3*.*2*	3	39.30	ZMM5636~ZMM5775	0.09	0.12	Y	Y		
*qCRR3*.*3*	3	52.30	ZMM2218~ZMM4682	0.05	0.10	Y	Y		
*qCRR3*.*4*	3	58.40	ZMM4682~ZMM5444	0.05	0.09			Y	
*qCRR5*.*1*	5	116.80	ZMM1155~ZMM0314	−0.04	0.04	Y	Y		Y
*qCRR8*.*1*	8	10.50	ZMM5060~ZMM5061	−0.04	0.05		Y	Y	
*qCRR8*.*2*	8	115.70	ID0041~ZM638	−0.04	0.05	Y	Y	Y	Y
*qCRR8*.*3*	8	123.70	ZM638~ZMM1682	−0.04	0.05	Y	Y	Y	Y
*qCRR9*.*1*	9	104.70	ZMM2323~ZMM0205	−0.05	0.08	Y	Y	Y	
*qCRR12*.*1*	12	53.80	ID0046~ID0133	−0.05	0.06	Y	Y		
*qCRR12*.*2*	12	89.80	ZMM0913~ZMM3752	−0.07	0.14	Y	Y		Y
*qCRR12*.*3*	12	106.10	ZMM3683~ZMM2365	−0.19	0.03			Y	
*qCRR13*.*1*	13	43.90	ZMM1307~ID0030	−0.04	0.04	Y	Y	Y	
*qCRR13*.*2*	13	73.50	ZMM2344~ZMM2343	−0.05	0.08				Y

*“Additive effect” indicates the estimated value for the genotype transmitted stably to offspring, and the “-” represents a negative contribution to disease. “R2” signifies the contribution rate of the locus to the phenotype; “Y” in the last four columns indicates that the QTL was detected at a specific trial site. YL, Yangluo; JX, Jinxian; LH, Luohe.

The 95% confidence intervals of the 14 mapped QTLs ranged from 0.8 to 25 cM; 60% were located within 5 cM (Table S3). Of these QTL, 2 loci (*qCRR8*.*2* and *qCRR8*.*3*) were detected in all four environments, and both were located in LG8. QTL *qCRR3*.*1*, *qCRR9*.*1*, *qCRR13*.*1*, *qCRR5*.*1*, and *qCRR12*.*2* were repetitively detected in three environments; the first three were found in Yangluo (2014 and 2015) and Jinxian (2015), and the last two were detected in Yangluo (2014 and 2015) and Luohe (2015) (Table 2). Five QTLs (*qCRR3*.*2*, *qCRR3*.*3*, *qCRR8*.*1*, *qCRR12*.*1*, and *qCRR12*.*2*) were detected in two environments. Locus *qCRR8*.*1* was associated with the *DI* from Yangluo and Jinxian in 2015, while the other four loci were found in Yangluo in 2014 and 2015. The loci.*qCRR3*.*4* and *qCRR12*.*3* were detected only in Jinxian, while *qCRR13*.*2* was detected only in Luohe.

**Figure 4:**
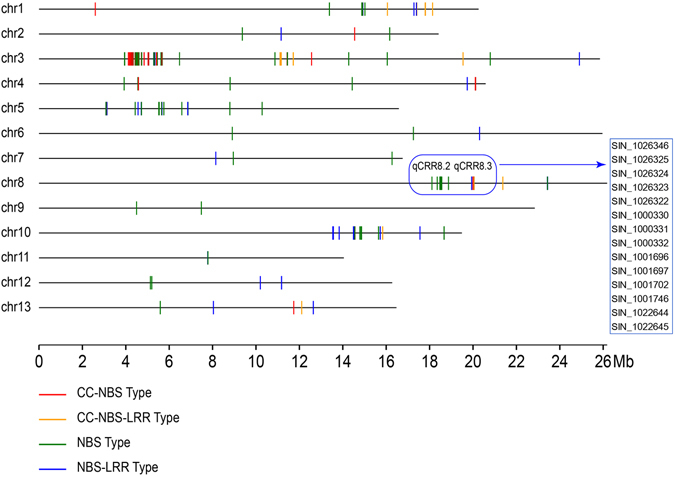
The predicted nucleotide-binding site (NBS)-encoding resistance genes in the *qCRR8.2* and *qCRR8.3* regions. Distributions of NBS-encoding resistance gene models (R-genes) along the sesame genome are denoted with short coloured lines. The 14 R-genes fell into the QTL listed in the box.

Sesame charcoal rot resistance-related QTLs were distributed differently among the LGs. LG3 contained the greatest number of mapped loci, with four QTL, followed by LG8 and LG13, with three QTLs each (Table 2). In LG12, the loci *qCRR12*.*2* had the highest phenotype contribution rate (14.16%). After mapping the flanking markers to the sesame genome^34^, the locus *qCRR12*.*2* fell into a region that included 15 genes. Annotation of these genes predicted a cluster of plant receptor-like serine threonine kinase (RLK) genes (SIN_1001382, SIN_1001381, SIN_1001380, SIN_1001379, SIN_1001377, SIN_1001376, SIN_1001373, SIN_1001372). It has been hypothesised that the RLK gene family expansion allowed accelerated evolution among domains implicated in signal reception, playing a central role in signalling during pathogen recognition^43^. We also focused on *qCRR8*.*2* and *qCRR8*.*3* for their common function in different environments. The physical mapping regions of the two loci on the sesame genome showed they coincidently contained several homologous plant disease resistance genes encoding nucleotide**-**binding sites (NBSs) (Fig. 4) ^44^. Genes encoding NBSs are the largest class of disease resistance genes in plants^44^. Thus, these mapped loci may represent an important locus for sesame resistance to charcoal rot disease.

## Conclusions

This study provided a novel genetic map for sesame and generated 424 polymorphic SSR markers. The mean interval between adjacent markers was 5.1 cM. Based on the constructed genetic map, 14 QTLs for sesame charcoal rot disease resistance were detected, with contribution rates of 3–14.16%; ~60% of these were located within 5 cM (95% confidence interval). The QTL with the highest phenotype contribution rate was *qCRR12*.*2*. Two loci (*qCRR8*.*2* and *qCRR8*.*3*) were detected in plants grown in all the four trial environments. QTL mapping revealed several candidate genes that were predicted to confer disease resistance. Thus, the genetic map of sesame is competent for mapping genes or QTLs. Moreover, the genetic map consisted of SSR markers, which will be more easily to be supplemented in a common molecular laboratory rather than SNP-based maps. Thus, this study will provide a useful reference for gene mapping and genetic studies in sesame.

## Methods

### Plant material

This study employed a RIL population (F8) with 548 lines generated from the cross between ZZM2748 (P1, male parent) and Zhongzhi No. 13 (P2). ZZM2748 is susceptible to sesame charcoal rot. Zhongzhi No. 13 showed high resistance to the disease, and it was *de novo* sequenced in 2014^34^. Both materials were preserved at the National Medium-term Sesame GenBank of China (Wuhan, China).

### Evaluation for charcoal rot response

As charcoal rot is a common disease in most sesame traditional planting areas, the natural responses of the RIL populations (ZZM2748 × Zhongzhi No. 13, F8:9) were evaluated in three typical sites that had suffered from *M*. *phaseolina*, including Luohe (2015), Jinxian (2015), and Yangluo (2014 and 2015), during the normal sesame planting season (June–September). In each field trial, the 548 lines were grown in a randomised complete block design with three replicate plots, each comprising three 2-m rows spaced 40 cm apart with a plant spacing of 10–20 cm. The highly susceptible material ZZM2748 was planted in every tenth plot as a check.

When over 50% of plants in each growing area exhibited apparent charcoal rot symptoms at the end stage of flowering, an investigation was performed on all plants having disease progressions of differing degrees (*I*) as follows: 0 = normal plant without disease spots; 1 = less than 1/3 of the plant and less than 1/4 of the capsules exhibited charcoal rot; 3 = 1/3 to 2/3 of the plant and 1/4 to 1/2 of the capsules exhibited charcoal rot; 5 = over 2/3 of the plant and 1/2 to 3/4 of the capsules exhibited charcoal rot; 7 = the entire plant died. The disease index (DI) was calculated based on the following formula^45^:

$$\begin{array}{c}D{I}_{N}=\sum _{i=1}^{n}({X}_{i}\times I)/(7\times \sum _{i=1}^{n}{X}_{i}),i=0,1,3,5,7\end{array}$$

(where *Xi* represents the plant number with disease degree *I*).

### SSR marker development

A Perl script MISA tool (http://pgrc.ipk-gatersleben.de/misa) was used to search microsatellite sites in the sesame genome (http://ocri-genomics.org/Sinbase) and transcriptome sequences^28, 34, 35, 38^. The SSRs with mono-, di-, tri-, tetra-, penta-, and hexa-nucleotide repeat units and compound units comprising two or more repeat motifs but interrupted by ≤100 bases were identified, and the minimum repeat numbers were defined as ten for mono-, six for di-, and five for tri-, tetra-, penta- and hexa-nucleotide repeat SSRs. Following the steps, the two Perl scripts p3_in.pl and p3_out.pl were used to handle the data generated from MISA and to format input data for primer design.

Based on the SSR flanking sequences, PRIMER3^46^ software was employed to design the primer pairs. The major parameters were adjusted as follows: primer length of 18–23 bases (optimal, 20 bases), GC content of 40–70% (optimal, 50%), annealing temperatures of 50–60 °C (optimal, 55 °C), and PCR product size of 100–400 bp (optimal, 200 bp). The markers developed based on sesame genome sequence were named with the prefix “ZMM”, “D”, or “ID” (genomic SSR), and those from transcriptomes were named with the prefix “ZM” (cDNA-SSR). All primer pairs were synthesised by GenScript Co., Ltd. (Nanjing, China).

### DNA extraction and PCR

At the early flowering stage, healthy young leaves of ZZM2748 and Zhongzhi No. 13, and the 548 RILs (F8) were selected and used for total genomic DNA extraction employing the cetyltrimethylammonium bromide (CTAB) method^32, 47^. DNA concentration qualities were estimated using an ND-1000 spectrophotometer (NanoDrop, Wilmington, DE, USA) at 260 nm, and the quality was confirmed by 0.8% agarose gel electrophoresis using a lambda DNA standard.

A 10-μl reaction mixture used for PCR contained 2.5 μl of template DNA (20 ng/ml), 0.2 μl of 5 mM/μl dNTP, 0.2 μl of 5 U/ml Taq polymerase (Bicolor, Shanghai, China), 0.45 μl of 18 pmol/μl of each primer, 1 μl of 10 × buffer, and 5.2 μl of ddH_2_O. PCR was performed on a thermocycle instrument (S1000, Bio-Rad, Hercules, CA, USA) using the following conditions: 10 min at 94 °C, then 30 cycles of 45 s at 94 °C, 45 s at 50−60 °C, and 1 min at 72 °C, followed by a final extension at 72 °C for 10 min. The PCR products were separated with 6% denaturing polyacrylamide gels and visualised after silver staining.

### Linkage map construction

The polymorphic SSR markers were used to genotype 548 lines. Using the software JoinMap 4^48^, the segregation ratios of these markers were evaluated using the chi-squared test, and significantly distorted (*P* < 0.01) markers were removed. With a logarithm of minimum odds (LOD) score of 4.0, the remaining markers were grouped and ordered according to their pair-wise recombination frequencies. The Kosambi mapping function was chosen to translate the recombination frequencies into map distances in cM. The goodness of fit of the calculated regression map for each tested position was checked with default parameters.

### QTL analysis

The frequency distributions of the mean phenotypic data for all RILs in each trial were analysed using R package software (https://www.r-project.org/). The QTLs related to charcoal rot resistance in sesame were detected with Windows QTL Cartographer 2.5 software (Microsoft, Inc., Redmond, WA, USA) using the composite interval mapping method. An associated peak with LOD score over 2.5 was judged as the presence of a QTL, and the statistical significance of the QTL effect was determined based on 1,000 permutations. The detected QTLs were named according to trait and LG location, referring to the rules of wheat gene nomenclature (http://wheat.pw.usda.gov/ggpages/wgc/98/Intro.htm).

## Electronic supplementary material

Supplementary Dataset 1
